# Micro-morphological study of *Evolvulus* spp. (Convolvulaceae): the old world medicinal plants

**DOI:** 10.1186/s40529-016-0141-y

**Published:** 2016-09-28

**Authors:** Kanapol Ketjarun, George W. Staples, Sasivimon C. Swangpol, Paweena Traiperm

**Affiliations:** 1 Department of Plant Science, Faculty of Science, Mahidol University, Rama VI Road, Ratchathewi, Bangkok, 10400 Thailand; 2 Herbarium, Royal Botanic Gardens, Kew, Richmond, Surrey, TW9 3AE UK

**Keywords:** *Evolvulus alsinoides*, *Evolvulus nummularius*, *Evolvulus glomeratus*, Leaf anatomy, Medicinal plant, Pollen, Seed coat

## Abstract

**Background:**

Several medicinal properties have been reported for plants in the genus *Evolvulus*, such as a brain tonic and antifungal from *Evolvulus alsinoides*, and a sedative and an anthelmintic from *Evolvulus nummularius*. Therefore, the correct identification of the source plants is critically important. The aim of this research was to investigate the micromorphology of two *Evolvulus* taxa used for herbal medicines compared with one worldwide ornamental species by using peeling, paraffin embedding, acetolysis, and SEM methods in order to support species identification.

**Results:**

Our findings indicate that all taxa share several common features, such as a single layer of epidermis on both sides of leaf surfaces, sinuous anticlinal epidermal cell walls, anomocytic, paracytic or laterocytic stomata, and capitate glandular trichomes. Y-shaped hairs were found in two species but not in *E. nummularius*. Similarly, isobilateral mesophyll occurs in both *E. alsinoides* and *Evolvulus glomeratus*, but a dorsiventral mesophyll is present in *E. nummularius*. Stems consist of a single layer of epidermis, one to four chlorenchyma layers, one to seven layers of cortical cells and a bicollateral bundle with pith in the center. The seed coat epidermal cell shapes were irregular or polygonal with raised and undulated anticlinal boundaries, and folded or flattened to concave periclinal walls. Pollens of all taxa are monads, spheroidally shaped with 28–47 µm diameter, and 15-pantocolpate apertures type with microechinate ornamentation.

**Conclusions:**

An identification key to species is constructed based on leaf anatomy and seed coat characters. This data can be used in other subjects such as pharmaceutical botany, organic chemistry, taxonomy and horticulture, in terms of species identification.

## Background

The genus *Evolvulus* L. belongs to the family Convolvulaceae with two taxa (*Evolvulus alsinoides* (L.) L. and *Evolvulus nummularius* (L.) L.) that are used in Asian herbal medicine (Auddy et al. 2003; Chen 2007; Khare 2007; Ayyanar and Ignacimuthu 2011; Naikawadi et al. 2016), while *Evolvulus glomeratus* Nees & Martius subsp. *grandiflorus* (Parodi) Ooststr. is a worldwide ornamental plant (Staples 2010). Some medicinal properties have been reported from *E. alsinoides* and *E. nummularius*. Several substances were found in *E. alsinoides*, i.e., flavonols, flavonoids, alkaloids, cardiac glycosides, saponins, the alkanes pentatriacontane and triacontane, the phytosterol, β-sitosterol, phenolics, and tannins (Austin 2008; Naikawadi et al. 2016) and the plant is used as brain and memory tonic herb (Naikawadi et al. 2016), an anti-asthmatic, for treating uterine bleeding (Khare 2007), insanity, epilepsy and nervous debility (Auddy et al. 2003), for antibacterial, antifungal, and antiulcer properties (Austin 2008). The most common uses for medicinal applications are from India and surrounding regions (Manandhar 1985; Auddy et al. 2003; Khare 2007; Ayyanar and Ignacimuthu 2011; Naikawadi et al. 2016), however uses from some other countries in Southeastern Asia were found such as Taiwan (Chen 2007), Vietnam, Thailand (Austin 2008), and Philippines (Quisumbing 1978). In the New World people also use *E. alsinoides*, but there are fewer reports than the Old. Moreover, weak sedative and anthelmintic properties were reported in *E. nummularius* (Khare 2007; Ayyanar and Ignacimuthu 2011). Because of all these attributed properties, two *Evolvulus* species have been sold commonly via the internet as a powder used for brewing medicinal tea. Therefore, a proper identification is needed to correctly identify herbal ingredients used in remedies sold for home use.

Plant anatomy is one of the alternative ways to differentiate plant species (Stuessy 2009). Previous research on vegetative anatomy in Convolvulaceae has been reported to be useful for classification within several genera such as *Argyreia* Lour. (Sayeedud-Din 1953; Tayade and Patil 2012b; Staples et al. 2015), *Calystegia* R.Br. (Tayade and Patil 2012b), *Cressa* L. (Tayade and Patil 2012b), *Evolvulus* L. (Tayade and Patil 2012b; Harms 2014), *Hewittia* Wight & Arn. (Tayade and Patil 2012b), *Hildebrantia* Vatke (Tayade and Patil 2012b), *Ipomoea* L. (Sayeedud-Din 1953; Tayade and Patil 2012b), *Merremia* Dennst. (Pisuttimarn et al. 2013), and *Quamoclit* Mill., (Sayeedud-Din 1953) which is now treated as a synonym of *Ipomoea* (Staples 2010). However, only one species of *Evolvulus*, *E. alsinoides,* has been investigated (Metcalfe and Chalk 1950; Tayade and Patil 2012b). Metcalfe and Chalk (1950) provided some diagnostic characters for the genus, such as two-armed or Y-shaped hair, cruciferous stomata type and cotyledons with secretory cells.

Hallier (1893) separated Convolvulaceae into two informal, non-taxonomic groups based on pollen type; Echinoconieae (grains with spines) and Psiloconiae (grains without spines, appearing ‘smooth’). However, Erdtman (1952) used different terms to separate Convolvulaceae pollen into two types: *Ipomoea* type and other types. Meanwhile, pollen morphology of some groups of Convolvulaceae has been studied such as Convolvulaceae in southern new world (Tellería and Daners 2003), *Bonamia* Thouars (Lewis 1971), *Convolvulus* L. (Perveen et al. 1989; Menemen and Jury 2002), *Cuscuta* L. (Hamed 2005; Costea et al. 2008; Welsh et al. 2010; Dettke et al. 2014), *Ipomoea* (Hsiao and Kuoh 1995; Rajurkar et al. 2011; Robertson 1982), *Odonellia* K.R. Robertson (Robertson 1982) and *Stylisma* Raf. (Lewis 1971). A recent study of South American Convolvulaceae pollen classified *Evolvulus* pollen into microechinate-microgranulate pollen (Tellería and Daners 2003) and *E. alsinoides* has oval and tricolpate type (Singh and Dhakre 2010).

Seeds also offer useful taxonomic data. Convolvulaceae seeds provided some taxonomically significant characters to construct keys to some species in Egypt (Abdel Khalik and Osman 2007) and also some *Ipomoea* species (Rao and Leela 1993). The seed characters used to construct a key to species are shape, seed ornamentation, anticlinal boundaries and epidermal cell shape (Gunn 1969; Rao and Leela 1993; Abdel Khalik and Osman 2007). Seed morphology of *Cuscuta* and *Ipomoea* were studied by several researchers (Rao and Leela 1993; Abdel Khalik 2006; Hamed 2005; Abdel Khalik and Osman 2007).

In this study, leaf and stem micromorphological characters of three taxa in the genus *Evolvulus* are investigated and compared. In addition, seed and pollen morphology were investigated to support taxonomic identification because some medicinal preparations use the whole plant thus pollen and/or seeds could be present as well as leaves and stems. This approach maximizes the capacity for making an identification of any plant parts obtained from herbal medicaments. Furthermore, this research highlights the need for anatomical and micromorphological investigation of *Evolvulus* in tropical America, where about 100 species are known to occur (Junqueira and Simão-Bianchini 2006). So far as we know, there has been no intensive anatomical study done for the neotropical taxa of *Evolvulus*.

## Methods

### Sample collection and herbarium specimen preparation

Fresh materials were collected and voucher specimens were prepared as a standard method for plant taxonomy (Bridson and Forman 1999), and deposited at the Forest Herbarium (BKF) and Queen Sirikit Botanic Garden Herbarium (QBG) (Table 1).

**Table 1:** List of specimens used in this study

Plant species	Localities	Collector number	Herbarium
*Evolvulus alsinoides* var. *decumbens*	Thailand, Khon Kaen	KK & PT 12	BKF, QBG
	Thailand, Phetchaburi	KK & PT 13	BKF, QBG
*E. glomeratus* ssp. *grandiflorus*	Thailand, Bangkok	KK & PT 11	BKF, QBG
	Thailand, Khon Kaen	KK & PT 14	BKF, QBG
*E. nummularius*	Thailand, Bangkok	KK & PT 10	BKF, QBG
	Thailand, Khon Kaen	KK & PT 15	BKF, QBG

### Anatomical study (peeling and paraffin methods)

Leaf surfaces from five leaves in each taxon were scraped by a razor blade, stained with 1 % Safranin-O and dehydrated by concentration series of ethanol. Leaf epidermal slides were mounted in DePeX mounting media. At least five leaves from each taxon were cut, dehydrated, infiltrated, and embedded in paraffin using a protocol modified from Johansen (1940). Sections were cut by sliding microtome (Leica SM2000R). Plant tissues were stained by Safranin-O and counterstained with Fast green and mounted in DePeX. Permanent slides were investigated and photographed under light microscopy using an Olympus BX43 with Olympus DP11 digital camera attached. The anatomical terminology used follows Metcalfe and Chalk (1950), Chen et al. (2008) and Pisuttimarn et al. (2013).

### Pollen morphological study

Anthers from dried flower buds were treated by standard acetolysis method (Erdtman 1960). For light microscopy (LM), pollen was preserved in silicone oil. At least fifty grains of pollen were investigated randomly under LM. Pollen for scanning electron microscopy (SEM) was dried in the air, and coated with platinum and palladium (Pt + Pd) by ion sputter (Hitachi E102). Photographs were taken under SEM (Hitachi S-2500). Pollen terminology according to an illustrated handbook (Hesse et al. 2009) was used to describe pollen features.

### Seed morphology

Mature seeds were fixed in 70 % ethanol and cleaned by sonicator. Samples were fixed on a stub, coated with platinum and palladium (Pt + Pd) by ion sputter (Hitachi E102) and investigated by SEM (Hitachi S-2500). Descriptions follow Abdel Khalik and Osman (2007) and Juan et al. (1999).

## Results

### Leaf and stem anatomy

**Fig. 1:**
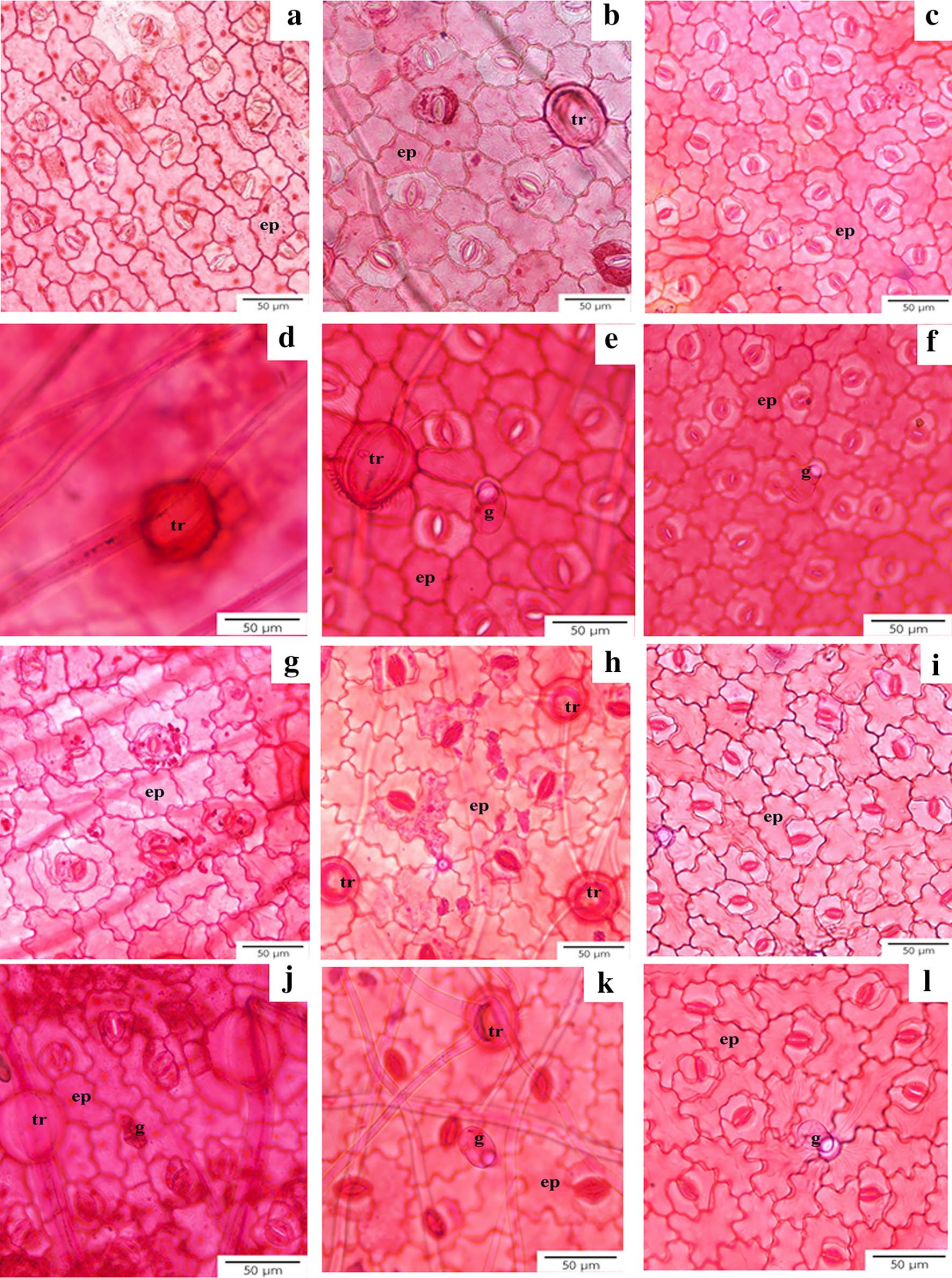
Epidermis under LM. Sinuous or straight to curved anticlinal wall and stomata occurring at the adaxial surface of *E. alsinoides* (**a**), *E. glomeratus* (**b**) and *E. nummularius* (**c**). Y-shaped hair on adaxial surface of *E. alsinoides* from Khon Kaen (**d**). Y-shaped hair and capitate gland of *E. glomeratus* (**e**), capitate gland on leaf surface of *E. nummularius* (**f**). Sinuous or straight to curved anticlinal walls on abaxial epidermal layer of *E. alsinoides* (**g**), *E. glomeratus* (**h**) and *E. nummularius* (**i**). *Evolvulus alsinoides* (**j**) and *E. glomeratus* (**k**) possess the Y-shaped hairs and capitate glands, but *E. nummularius* (**l**) has no hairs. *ep* epidermis, *tr* trichome, *g* capitate gland

Leaf epidermal cell shapes were polygonal with sinuous or straight to curved anticlinal walls (Fig. 1a–c, g–i). Anisocytic, anomocytic, and paracytic stomatal types were found on both leaf surfaces, especially on the abaxial side (Fig. 1g–i). Y-shaped hairs were present on both leaf surfaces of *E. alsinoides* collected from Khon Kaen and *E. glomeratus*, and the abaxial side of *E. alsinoides* from Phetchaburi (Fig. 1d, e, h–k). However, the hairs were absent in *E. nummularius* (Fig. 1c, f, i, l). Capitate glands were also present on both surfaces (Fig. 1e, f, j–l). The glands are composed of one small base cell, a single short stalk cell and two to four apical cells in a spheroidal or ellipsoid head (Fig. 1j, k).

#### Leaf

**Fig. 2:**
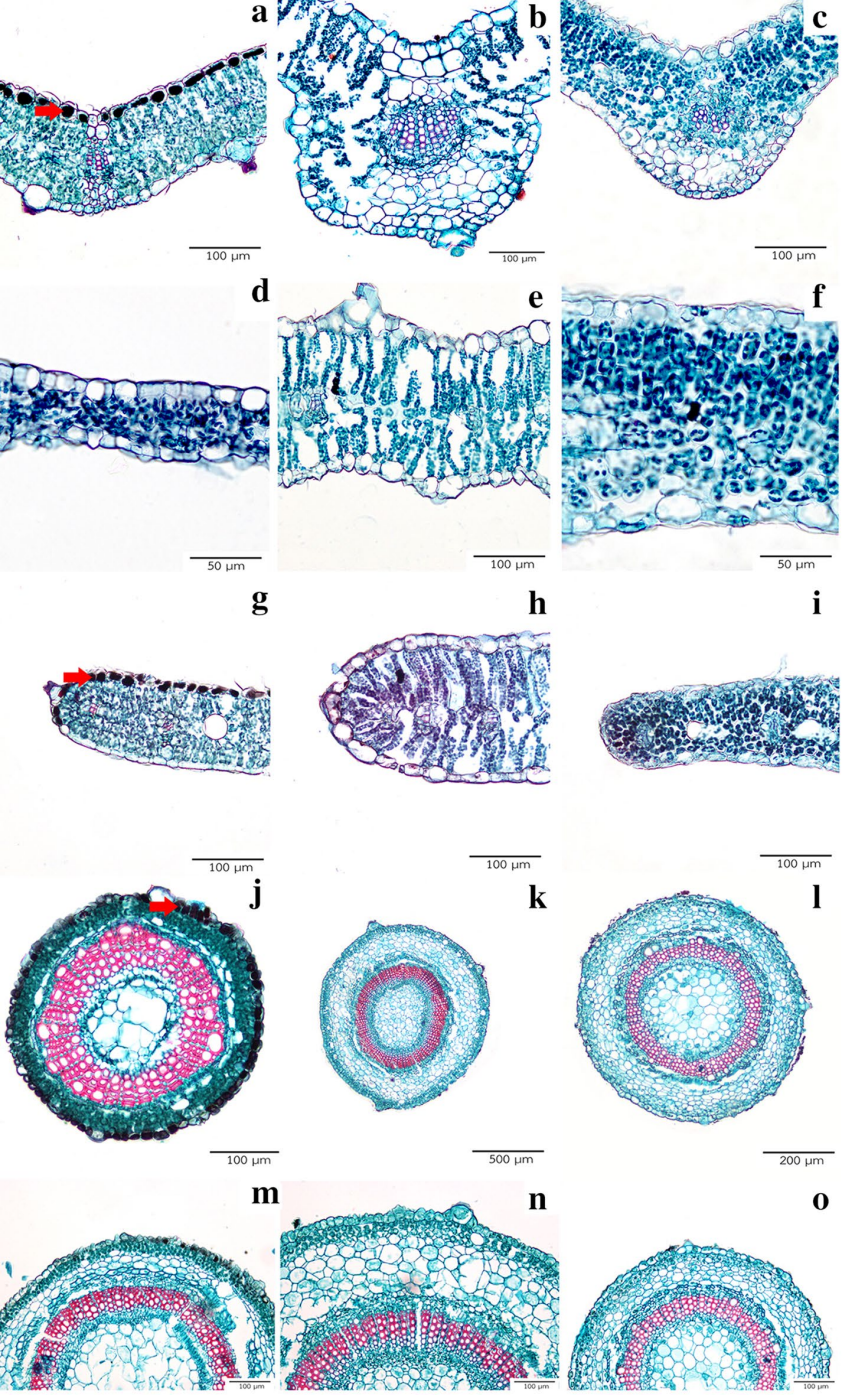
Transverse sections of leaf and stem. A single epidermal layer and bicollateral vascular bundle of *E. alsinoides* (**a**), *E. glomeratus* (**b**) and *E. nummularius* (**c**). Tanniniferous epidermal cell is unique in *E. alsinoides* from Petchaburi (**a**, **g**, **j** indicated by *red arrows*). Isobilateral mesophyll present in *E. alsinoides* (**d**) and *E. glomeratus* (**e**). *Evolvulus nummularius* showing dorsiventral mesophyll type (**f**). Rounded leaf margins of *E. alsinoides* (**g**), *E. glomeratus* (**h**) and *E. nummularius* (**i**). Outline of stem in *E. alsinoides* (**j**), *E. glomeratus* (**k**) and *E. nummularius* (**l**). Enlarged view to show the stem layers of *E. alsinoides* (**m**), *E. glomeratus* (**n**) and *E. nummularius* (**o**)

Blades in transverse section presented a single epidermal layer on both sides. Tanniniferous epidermal cells were detected only on the adaxial epidermis of *E. alsinoides* from Phetchaburi (Fig. 2a). Isobilateral mesophyll type was detected in *E. alsinoides* and *E. glomeratus*, while dorsiventral mesophyll type was restricted to *E. nummularius* (Fig. 2d–f). Leaf thickness is between 70–140 µm, except in *E. glomeratus:* its leaves are thicker than the others with approximately 200–240 µm thickness (Fig. 2d–f). Vascular bundles at the midrib of all species are bicollateral type consisting of the sclerenchyma arch (Fig. 2a–c). Leaf margins are rounded to slightly acute (Fig. 2g–i). Leaf characters of all species are summarized in Table 2.

**Table 2:** Comparison of the leaf anatomy in three taxa

Plant taxa	*E. alsinoides* var. *decumbens* (KK & PT 12)	*E. alsinoides* var. *decumbens* (KK & PT 13)	*E. glomeratus* ssp. *grandiflorus*	*E. nummularius*
Characters				
Leaf epidermis				
Adaxial surface				
Cell shape	Polygonal	Polygonal	Polygonal	Polygonal
Anticlinal cell wall	Sinuous or straight to curved	Sinuous or straight to curved	Sinuous or straight to curved	Sinuous or straight to curved
Stomatal type	Anisocytic, anomocytic and paracytic	Anisocytic, anomocytic and paracytic	Anisocytic, anomocytic and paracytic	Anisocytic, anomocytic and paracytic
Y-shaped hairs	Present	Absent	Present	Absent
Capitate gland	Present	Present	Present	Present
Abaxial surface				
Cell shape	Polygonal	Polygonal	Polygonal	Polygonal
Anticlinal cell wall	Sinuous or straight to curved	Sinuous or straight to curved	Sinuous or straight to curved	Sinuous or straight to curved
Stomatal type	Anisocytic, anomocytic and paracytic	Anisocytic, anomocytic and paracytic	Anisocytic, anomocytic and paracytic	Anisocytic, anomocytic and paracytic
Y-shaped hairs	Present	Present	Present	Absent
Capitate gland	Present	Present	Present	Present
Leaf transverse section				
Epidermal layer	Single layer	Single layer	Single layer	Single layer
Parenchyma at midrib	2‒4 layers	2‒4 layers	4‒5 layers	3‒7 layers
Mesophyll	Isobilateral	Isobilateral	Isobilateral	Dorsiventral
Midrib vascular bundle	Bicollateral type	Bicollateral type	Bicollateral type	Bicollateral type
Leaf thickness (µm)	70‒100	70‒100	200‒240	120‒140
Leaf margins	Rounded to slightly acute	Rounded to slightly acute	Rounded to slightly acute	Rounded to slightly acute
Stem transverse section				
Epidermal layer	Single layer	Single layer	Single layer	Single layer
Chlorenchyma layer	1‒3 layers	2‒4 layers	2‒4 layers	2‒4 layers
Cortex	1‒3 layers	3‒5 layers	5‒7 layers	5‒7 layers
Vascular bundle	Bicollateral type	Bicollateral type	Bicollateral type	Bicollateral type
Pith	Present	Present	Present	Present

#### Stems

As seen in transverse sections stems in all species are composed of a single epidermis with one to four layers of chlorenchyma cells. *Evolvulus alsinoides* is the only species that possesses the tanniferous cells in the epidermis. Cortex was restricted by one to seven parenchyma cell layers. Vascular tissues are the bicollateral bundle type with cylindrical xylem (Fig. 2j–o). Pith was observed at the center. All stem anatomical characters are summarized (Table 2).

### Pollen morphology

**Fig. 3:**
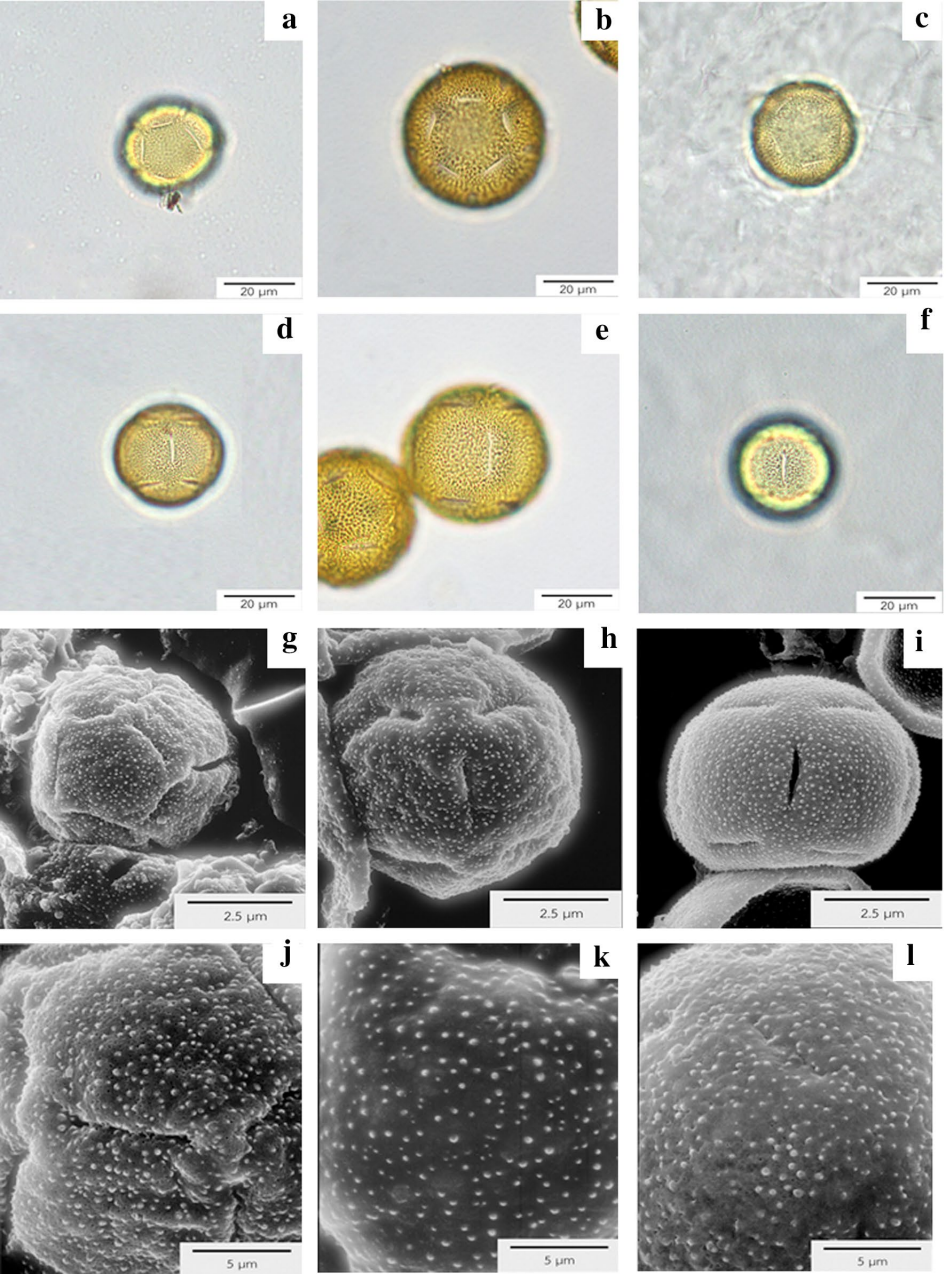
Pollen under light microscopy (LM) and scanning electron microscopy (SEM). **a**–**c** Polar view under LM of *E. alsinoides* (**a**), *E. glomeratus* (**b**) and *E. nummularius* (**c**), which demonstrates the monad pollen unit and 5-pantocolpate apertures. **d**–**f** Monad pollen under LM with 5-pantocolpate apertures at equatorial view of *E. alsinoides* (**d**), *E. glomeratus* (**e**) and *E. nummularius* (**f**). **g**–**i** SEM of *E. alsinoides* (**g**) in polar view, showing 5-pantocolpate apertures. Polar view under SEM of *E. glomeratus* (**h**), equatorial view of *E. nummularius* (**i**) presenting 5-pantocolpate apertures. **j**–**l** Microechinate ornamentation of *E. alsinoides* (**j**), *E. glomeratus* (**k**) and *E. nummularius* (**l**)

All species share some common pollen characters i.e., monads, medium size and 15-pantocolpate apertures (5 polar × 5 equatorial × 5 polar arrangement of the colpi) (Fig. 3a–i). The length of polar axis (P) is between 27–45 µm, whereas 28–47 µm in equatorial axis (E). Therefore, the P/E ratio is calculated to be a spheroidal shape. *Evolvulus alsinoides* and *E. nummularius* have similar size; 27–33 µm in polar axis and 28–33 µm in equatorial axis: however *E. glomeratus* differs from the other two species by having 39–45 µm polar axis and 41–47 µm equatorial length. Pantocolpate apertures and microechinate ornamentation were recorded in all species (Fig. 3j–l) (Table 3).

**Table 3:** Pollen and seed morphological characters in all taxa

Plant taxa	*E. alsinoides* var. *decumbens* (KK & PT 12)	*E. alsinoides* var. *decumbens* (KK & PT 13)	*E. glomeratus* ssp. *grandiflorus*	*E. nummularius*
Characters				
Pollen morphology				
Pollen unit	Monad	Monad	Monad	Monad
Size polar axis (P) (µm)	29–32	27–33	39–45	28–33
Size equatorial axis (E) (µm)	28–31	29–33	41–47	28–33
Shape	Spheroidal	Spheroidal	Spheroidal	Spheroidal
Aperture	15-pantocolpate	15-pantocolpate	15-pantocolpate	15-pantocolpate
Ornamentation	Microechinate	Microechinate	Microechinate	Microechinate
Seed morphology				
Shape	Ovoid	Ovoid	No material available	Broadly elliptic
Size	1–1.5 × 1–1.5	1–1.5 × 1–1.5	No material available	1.5–2 × 1–1.5
Color	Yellow/orange/red/brown	Yellow/orange/red/brown	No material available	Yellow/brown
Surface	Glabrous	Glabrous	No material available	Glabrous
Epidermal cell shape	Irregular, polygonal	Irregular, polygonal	No material available	Irregular, polygonal
Anticlinal boundaries	Undulate	Undulate	No material available	Undulate
Periclinal cell wall	Folded	Folded	No material available	Flattened to concave

### Seed morphology

**Fig. 4:**
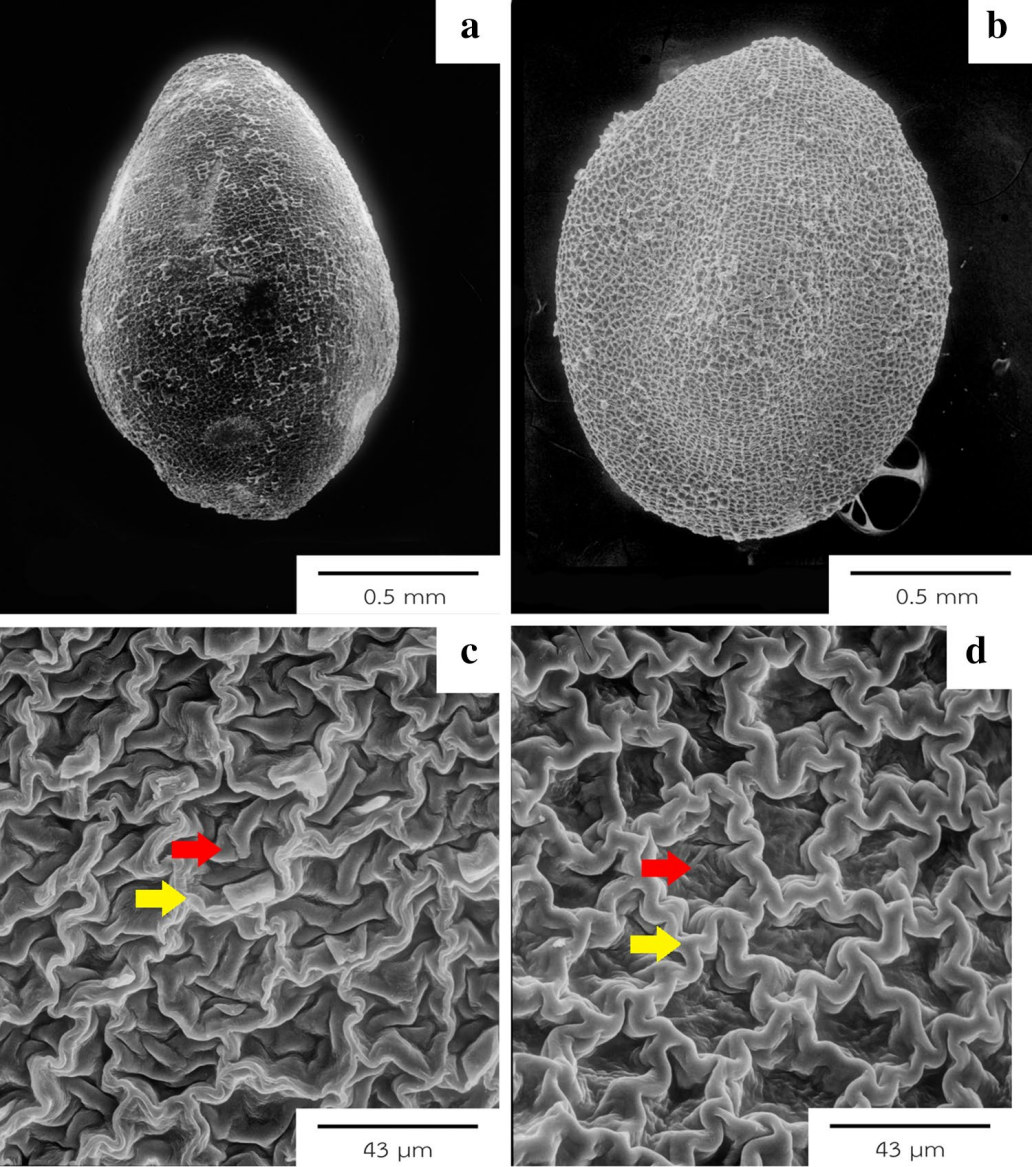
Seed morphology from SEM. Glabrous and ovoid shaped seed of *E. alsinoides* (**a**) from Petchaburi, broadly ellipsoid seed with glabrous surface in *E. nummularius* (**b**), close up of *E. alsinoides* (**c**) from Khon Kaen, seed testa showing undulate anticlinal boundaries (*yellow arrows*) and folded periclinal cell wall (*red arrows*). *Evolvulus nummularius* (**d**) displayed undulate anticlinal boundaries with flat to concave periclinal cell wall

Seeds of two species studied (the seeds of *E. glomeratus* were not available) varied from 1–2 mm in length and 1–1.5 mm in width with yellow, orange, red or brown seed coat (Table 3). The seeds are glabrous. Seed shape was trigonous-ovoid with glabrous surface. Epidermal cell shapes were irregular or polygonal in both species with undulate and raised anticlinal boundaries. Periclinal cell walls were flattened to concave in *E. nummularius* and folded in *E. alsinoides*. All seed features are summarized in Table 3 (Fig. 4).

## Discussion

This study provides for the first time comprehensive micro-morphological information for three *Evolvulus* taxa. Leaf and stem anatomical characters basically coincide with the descriptions of other Convolvulaceae species previously studied by Metcalfe and Chalk (1950).

General diagnostic characters for *Evolvulus* leaves on surface view are the sinuous or straight to curved anticlinal walls, anisocytic, anomocytic and paracytic stomatal types, the presence of Y-shaped hairs, and capitate trichomes. The presence of the anisocytic (or cruciferous) stomatal type corresponds to the previous report by Metcalfe and Chalk (1950). Most species have Y-shaped hairs on both sides of the leaves, except in *E. nummularius*. Furthermore, *E. alsinoides* from Phetchaburi has Y-shaped hairs on the abaxial surface only. The occurrence of Y-shaped hairs on both leaf surfaces was similar to the discovery of Metcalfe and Chalk (1950), and also matched with the study of Harms (2014) which recorded Y-shaped hairs in three *Evolvulus* species found in Texas and New Mexico. The function of Y-shaped hairs might be functionally related to reduction in water loss for the plant because *E. alsinoides* is always found on limestone in a habitat which has intense insolation accompanied by low humidity and dry soils. These non-glandular trichomes cover up the guard cells in order to protect the plant from dessication. Metcalfe and Chalk (1950) mentioned the secretory cells found in the cotyledons of many genera including *Evolvulus*, but did not specify the type of cell. Capitate glandular trichomes were found in all three *Evolvulus* taxa, and here we describe this character in detail for the first time.

The characters of leaf transverse sections useful for species separation are as follows: the presence of tanniniferous epidermal cells; type of palisade mesophyll and leaf thickness. *Evolvulus alsinoides* from Phetchaburi is the only plant sample that presented a storage substance on the adaxial epidermal cells, coincident with Metcalfe and Chalk (1950) treatment. Thai *Evolvulus* taxa showed both isobilateral and dorsiventral mesophyll types. Isobilateral type was found in *E. alsinoides* and *E. glomeratus*, while the dorsiventral type occurs in *E. nummularius*. Tayade and Patil (2012b) described a dorsiventral mesophyll for *E. alsinoides* from India, which is not congruent with our findings. The reason might be the differences between two populations of *E. alsinoides* from India and Thailand in terms of variability of anatomical features. It is also possible that Tayade and Patil (2012b) had another species entirely: there were no voucher specimens cited for the plants they studied, and thus we cannot be certain that our results are comparable with their findings.

According to the monograph by van Ooststroom (1934), *E. alsinoides* was separated into 15 varieties. It is possible that *E. alsinoides* collected from Phetchaburi might not be the same variety as the plant from Khon Kaen province as suggested by the differences of Y-shaped hairs as well as the storage epidermal cells and the mesophyll arrangement. Another possibility would be the environmental differences in two populations of *E. alsinoides* because *E. alsinoides* (KK & PT 12) came from limestone area but *E. alsinoides* (KK & PT 13) was collected from weedy place. The occurrence of Y-shaped hairs and dorsiventral mesophyll can clearly be used to separate *E. nummularius* from the others, while the other two species can be identified by leaf thickness. *Evolvulus alsinoides* has thinner leaves (70–100 µm) than *E. glomeratus* (200–240 µm). With sufficient fertilizer and water, cultivated plants are usually bigger than wild plants of the same species (Lau and Stephenson 1994) almost in all aspects as shown in leaf thickness from this study. Thus, a key to the species provided here has to be used with caution because the anatomical characters might be different from wild species. Presence of Y-shaped hair was also useful to distinguish the species of *Evolvulus* as was proposed recently by Harms (2014).

Stem anatomical characters are similar in all species; however, the stem diameter in *E. glomeratus* is bigger than the other two species. The presence of tanniferous and chlorenchyma cells in the stem has never been described previously. A bicollateral bundle is the typical type for the family Convolvulaceae as was formerly reported by Metcalfe and Chalk (1950) and Tayade and Patil (2012a).

Pollen characters of all taxa are quite similar in all aspects; therefore pollen morphology has low taxonomic value to identify *Evolvulus* taxa. The only one quantitative datum that can be used to separate *E. glomeratus* from other taxa was the pollen size. *Evolvulus glomeratus* possessed the largest pollen with 39–45 µm in polar axis (length) and 41–47 µm in equatorial axis (width). Pollen ornamentation and apertures are consistent with the investigation of Tellaría and Daners (2003) which classified *Evolvulus* pollen into Type II with microechinate exine. In their study, three species of *Evolvulus* were examined i.e., *E. arizonicus* A. Gray, *E. glomeratus* Nees & Mart. and *E. sericeus* Sw., therefore this investigation confirms the exine pattern of *Evolvulus* by having studied two more species in the genus. However, this current study disagrees with the reproductive biological examination of medicinal plant by Singh and Dhakre (2010) which recorded *E. alsinoides* pollen as tricolpate apertures and reticulate ornamentation. The photos of live plants shown in their paper looked quite different from *E. alsinoides* collected from Thailand. Sarma et al. (2007) have suggested that the pollinator of *E. nummularius* in India is a Graceful Awl snail (*Lamellaxis gracile*) on rainy days, while a honey bee (*Apis cerana indica*) is the pollinator on a sunny day. However, *E. nummularius* is not a native species in India. Therefore, the pollination relationships may not be typical for the native habitat in South America. Singh and Dhakre (2010) mentioned the honey bee as one of nine insect pollinators of *E. alsinoides*. From plant morphology, a snail should not be a pollinator for *E. alsinoides* and *E. glomeratus* because their flower position is not located close to the soil surface as it is for *E. nummularius*. Therefore, the honey bee and flies might be the pollinators of *E. alsinoides* and *E. glomeratus*.

Seed characters have taxonomic significance and can be used to construct a key to species. Periclinal walls differ between two taxa: folded in *E. alsinoides* and flat to concave in *E. nummularius*. The testal periclinal cell wall corresponded to that described by Abdel Khalik and Osman (2007).

## Conclusions

From leaf anatomical characters, there are some taxonomic aspects to distinguish these three *Evolvulus* taxa, therefore a key to taxa based on leaf anatomy was constructed.

Seed characters have taxonomic significance and can be used to construct a key to species.

This data serves as a base line to be used in other subjects such as taxonomy, horticulture, pharmaceutical botany and organic chemistry in terms of species identification, ornamental plant improvement and medicinal plant identification, respectively.
