# Erratum to: Micro-morphological study of *Evolvulus* spp. (Convolvulaceae): the old world medicinal plants

**DOI:** 10.1186/s40529-016-0144-8

**Published:** 2016-10-18

**Authors:** Kanapol Ketjarun, George W. Staples, Sasivimon C. Swangpol, Paweena Traiperm

**Affiliations:** 1 Department of Plant Science, Faculty of Science, Mahidol University, Rama VI Road, Ratchathewi, Bangkok, 10400 Thailand; 2 Herbarium, Royal Botanic Gardens, Kew, Richmond, Surrey, TW9 3AE UK

## Erratum to: Bot Stud (2016) 57:25 DOI 10.1186/s40529-016-0141-y

In the publication of this article [1], there was an error in the Abstract section which was published with incorrect pollen size results. The error: ‘Pollens of all taxa are monads, spheroidally shaped with 28–457 µm diameter, and 15-pantocolpate apertures type with microechinate ornamentation.’ Should instead read: ‘Pollens of all taxa are monads, spheroidally shaped with 28–47 µm diameter, and 15-pantocolpate apertures type with microechinate ornamentation.’ This has now been updated in the original article [1].
